# Neural correlates of grasping

**DOI:** 10.3389/fnhum.2014.00686

**Published:** 2014-09-09

**Authors:** Luca Turella, Angelika Lingnau

**Affiliations:** ^1^Center for Mind/Brain Sciences (CIMeC), University of TrentoTrento, Italy; ^2^Department of Cognitive Sciences, University of TrentoTrento, Italy

**Keywords:** prehension, grasping, reaching, fMRI, neurophysiology, motor system

## Abstract

Prehension, the capacity to reach and grasp objects, comprises two main components: reaching, i.e., moving the hand towards an object, and grasping, i.e., shaping the hand with respect to its properties. Knowledge of this topic has gained a huge advance in recent years, dramatically changing our view on how prehension is represented within the dorsal stream. While our understanding of the various nodes coding the grasp component is rapidly progressing, little is known of the integration between grasping and reaching. With this Mini Review we aim to provide an up-to-date overview of the recent developments on the coding of prehension. We will start with a description of the regions coding various aspects of grasping in humans and monkeys, delineating where it might be integrated with reaching. To gain insights into the causal role of these nodes in the coding of prehension, we will link this functional description to lesion studies. Finally, we will discuss future directions that might be promising to unveil new insights on the coding of prehension movements.

## Introduction

The capacity to reach and grasp objects, i.e., prehension, is at the basis of our daily interactions with objects. Prehension entails two main components: transport, i.e., reaching an object with the hand, and grasping, i.e., the preshaping of the hand with respect to the object’s intrinsic properties (e.g., shape and size). Previous monkey neurophysiological and human neuroimaging studies demonstrated that planning and execution of this complex skilled behavior, and of its two components, are encoded within specific neural substrates: the “prehension” network (Jeannerod, 1981; Jeannerod et al., 1995; Brochier and Umiltà, 2007; Castiello and Begliomini, 2008; Filimon, 2010; Grafton, 2010; Davare et al., 2011).

This Mini Review is thought as a brief introduction and as an update of two recent reviews on this topic (Filimon, 2010; Grafton, 2010). Here, we will focus on grasp coding and on its integration with reaching, as reaching has already been covered in recent contributions (Crawford et al., 2011; Vesia and Crawford, 2012). We will focus on a description of the role of the dorsal stream in grasp coding, despite recent investigations pointing to a possible involvement of the ventral stream in prehension (Verhagen et al., 2008, 2012). Throughout the review, we will touch the following main questions, which are still matter of investigation: (i) *where* the prehension system codes the two components; (ii) which regions are *necessary* for their coding; and (iii) at which stage they are possibly *integrated*.

In the first part, we will provide an anatomical and functional description of the prehension system in monkeys and humans. In the second part, we will describe lesion studies which allow drawing causal inferences on the role of the regions within the prehension system. In the last part, we will cover recent advances on grasp coding with a focus on the temporal aspects which we consider fundamental for obtaining new insights on the neural basis of prehension.

## Anatomical and functional description of the prehension system

The classical description of the monkey prehension system was based on the definition of a series of parallel cortico-cortical pathways connecting regions within the posterior parietal cortex (PPC) with regions of the frontal cortex possessing similar response properties. These pathways are considered crucial in the sensorimotor processing for the *planning* and *online control* of reaching, grasping and saccadic eye movements (Rizzolatti et al., 1998; Andersen and Buneo, 2002).

**Figure 1:**
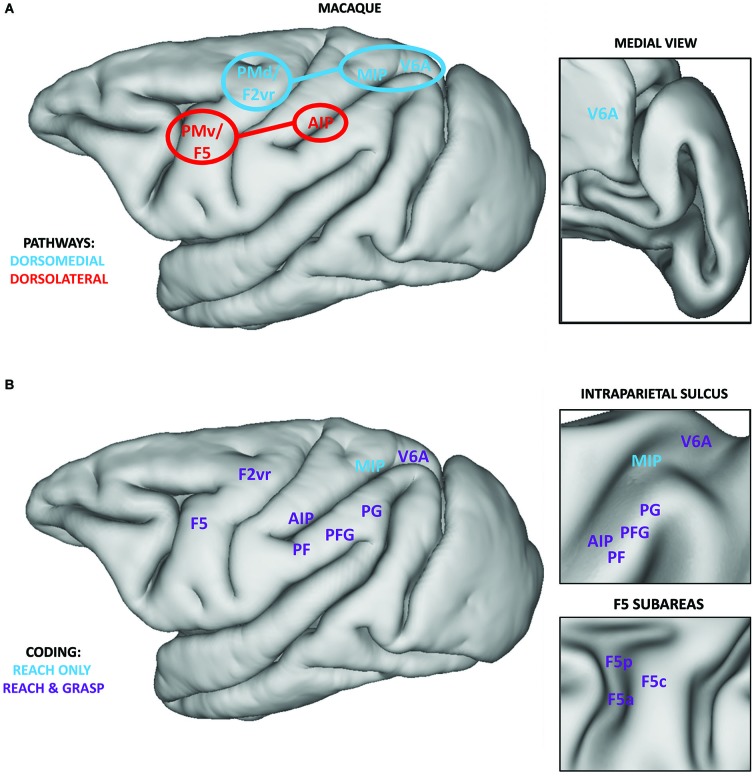
**(A)** Classical localization of core regions within the dorsomedial (blue) and dorsolateral pathways (red) overlaid on the lateral view of a macaque brain. Regions within the SPL (MIP and V6A) target the PMd (area F2vr), whereas AIP mainly targets F5, and its subarea F5p (Matelli and Luppino, 2001; Tanné-Gariépy et al., 2002; Rizzolatti and Matelli, 2003). Connections between the PPC and premotor cortices are highlighted. Within the inset, the position of area V6A on the medial surface of the macaque brain is shown. **(B)** Definition of regions within the PPC and premotor cortices showing coding for grasping and reaching (purple) or only for reaching (blue). Data for reach coding are extracted from a recent review (Battaglia-Mayer et al., 2006) and the results of a recent neurophysiological study (Lehmann and Scherberger, 2013). Data for grasp coding are extracted from various sources (Brochier and Umiltà, 2007; Rozzi et al., 2008; Fattori et al., 2010, 2012). Within the upper inset, the position of regions within the intraparietal sulcus is represented on an inflated brain surface. Within the lower inset, the position of the subareas of region F5 is represented on an inflated brain surface. Medial regions, except V6A, are not reported.

According to the classical model of prehension, the dorsolateral pathway is coding grasping, i.e., different grip types, whereas the dorsomedial pathway is coding reaching, i.e., information related to the transport phase (Figure 1A; Jeannerod et al., 1995; Caminiti et al., 1998; Culham et al., 2003; Culham and Valyear, 2006).

The dorsolateral pathway connects two core regions: the anterior part of the intraparietal sulcus (AIP; Murata et al., 2000; Baumann et al., 2009) within the inferior parietal lobule (IPL) and area F5 within the ventral premotor cortex (PMv; Rizzolatti et al., 1988; Murata et al., 1997; Raos et al., 2006; Fluet et al., 2010). This pathway has been classically described to be involved in visually guided grasping via the transformation of intrinsic properties of the to-be-grasped object into appropriate motor commands for hand pre-shaping (Jeannerod et al., 1995; Brochier and Umiltà, 2007). The neurophysiological basis of this sensorimotor transformation might be supported by visuomotor neurons (“canonical” neurons) described in AIP (Murata et al., 2000) and F5 (subareas F5p and F5c, Bonini et al., 2014) which are active while performing a grasping movement and while observing graspable objects. Most of these neurons show a strict congruence between the coded grip and the intrinsic properties of the object eliciting their visual response.

The dorsomedial pathway connects two regions within the PPC, area V6A (Bosco et al., 2010) and MIP (Johnson et al., 1996), with the dorsal premotor cortex (PMd; Caminiti et al., 1991). This pathway has been classically considered as coding reaching information for planning and controlling arm position during the transport phase, via the integration of somatosensory and visual information (Rizzolatti et al., 1998).

This initial model has been shown to be incomplete, as many neurophysiological investigations described neural activity related to both components of prehension within both pathways (Figure 1B). With respect to grasping, the IPL convexity (particularly area PFG), having direct connections to F5, seems to be critically involved in planning and executing grasping (Rozzi et al., 2008; Bonini et al., 2011, 2012). Core regions of the dorsomedial pathway, V6A and PMd (area F2vr), are coding not only reach, but also grasp-related information (Raos et al., 2004; Fattori et al., 2010, 2012). Similarly, many regions within both pathways are also involved in coding reaching (see Figure 3 in Battaglia-Mayer et al., 2006). Remarkably, even the core nodes of the grasp-related pathway (F5 and AIP) host neural populations coding reaching and even populations coding both reach and grasp information (Lehmann and Scherberger, 2013). Nevertheless, few other studies investigated the coding of both components within the same neural population (e.g., PMd and PMv, Stark et al., 2007). Consequently, it is difficult to assess, at least from a *functional* point of view, to which degree grasping and reaching are encoded independently, and at which stage they are integrated.

**Figure 2:**
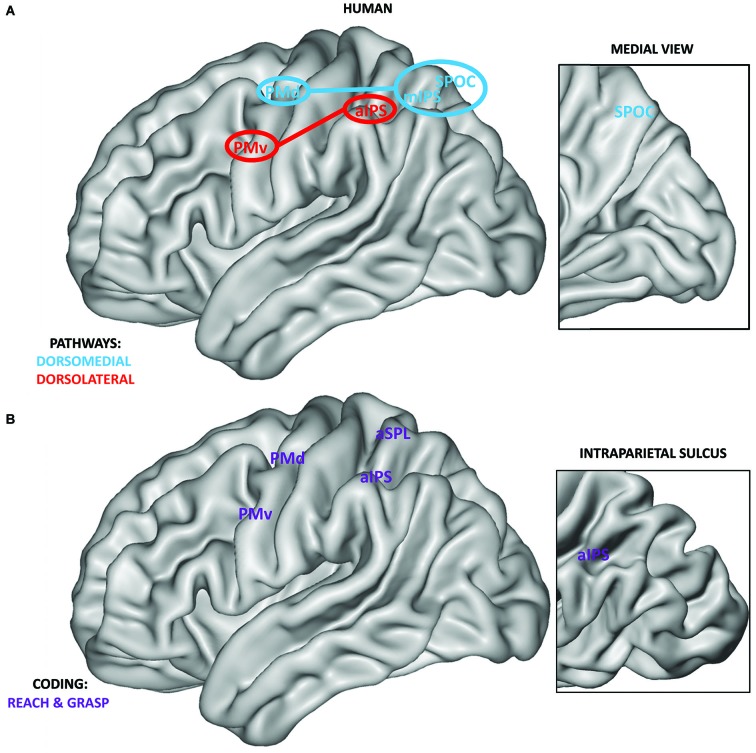
**(A)** Anatomical localization of human grasping regions within the dorsomedial (blue) and dorsolateral pathways (red). Connections between the PPC and premotor cortices are highlighted. As in monkeys, human PPC regions of the SPL are connected mainly with the PMd, whereas regions of the IPL are connected with the PMv (Tomassini et al., 2007). Within the inset, the position of SPOC on the medial surface of the human brain is shown. Medial regions, except for SPOC, are not reported. **(B)** Definition of regions within the PPC and premotor cortices showing grasp and reach coding (purple). Regions are extracted from the recent study by Fabbri et al. (2014) adopting a searchlight MVP analysis approach, i.e., covering the entire brain surface. Within the inset, the position of aIPS within the intraparietal sulcus is highlighted.

Monkey neurophysiological investigations provided the starting point for the definition of a similar human system via neuroimaging techniques which lack the high spatial and temporal resolution of neurophysiological recordings, but sample the whole brain, instead of only one or few nearby regions. The classical method for fMRI analysis adopts a univariate comparison of activity between different conditions for every single voxel. Using a univariate approach, a potential homologous prehension system has been described within the human PPC and premotor cortices (Culham et al., 2006; Culham and Valyear, 2006; Filimon, 2010; Figure 2A). With respect to the dorsolateral pathway, a possible homology was found for a region of the anterior intraparietal sulcus (aIPS; Culham et al., 2003; Frey et al., 2005) and for PMv (Cavina-Pratesi et al., 2010b), both recruited during grasping. Regarding the dorsomedial pathway, homologous reach-related areas were localized within the medial intraparietal sulcus (mIPS; Prado et al., 2005; Filimon et al., 2009), the superior parietal occipital cortex (area SPOC), the precuneus (Connolly et al., 2003; Prado et al., 2005; Filimon et al., 2009; Cavina-Pratesi et al., 2010b) and PMd (Filimon et al., 2007, 2009).

Univariate analyses also showed activity within the whole prehension network when comparing reaching only (Filimon et al., 2007, 2009) or reach-to-grasp movements (Culham et al., 2003; Turella et al., 2009) with respect to a baseline or control condition, resembling the widespread coding of both components of prehension shown in monkey. Whereas univariate analyses can identify areas in which either the reach or the grasp component leads to a higher overall signal, this approach does not allow drawing conclusions about the properties coded in these regions.

Recent advances in fMRI analysis permitted a more fine grained investigation of the properties of the prehension network by adopting Multivariate Pattern (MVP) analysis. Instead of carrying out massive univariate analysis separately for each voxel, this approach uses the pattern of activation over multiple voxels (Kriegeskorte and Bandettini, 2007). Recently, Gallivan et al. (2011, 2013) distinguished using MVP analysis between visually guided reach-to-grasp and reach-only movements (during planning and execution) within a number of predefined regions of the two pathways such as PMv, PMd, aIPS, mIPS and SPOC.

Similar results were obtained in a recent study using MVP analysis investigating the execution of non-visually guided actions (Fabbri et al., 2014). This study manipulated both grip type (i.e., whole hand grip vs. precision grip) and movement direction within the same paradigm. The results showed overlapping regions coding grasping and reaching within the whole prehension system (PMv, inferior PMd, anterior SPL, aIPS, see Figure 2B) and hint at a possible interaction between both types of coding within a subset of these regions (PMv, aIPS, anterior SPL).

To summarize, converging evidence from neurophysiological and neuroimaging studies suggests that, from a *functional* perspective, the strict subdivision of the prehension system in two independent pathways is not tenable as grasping seems to be coded, and possibly integrated with reaching, within both pathways.

## Lesion studies

Neurophysiological and neuroimaging methods are correlational by nature. Consequently, measuring grasp-related activity within a specific region does not prove its causal involvement in determining grasping at a behavioral level. Lesion studies provide fundamental information for the interpretation of neurophysiological and neuroimaging data.

A number of monkey lesion studies (Battaglini et al., 2002; Hwang et al., 2012; Yttri et al., 2014; for a review, see Andersen et al., 2014) showed that the so-called Parietal Reach Region, comprising V6A, MIP and area 5v (Andersen et al., 2014), is causally involved in the planning and online control of reaching. After resection of V6A, monkeys were unable to correctly perform object-directed prehension movements, not only misreaching targets but showing also grasping deficits, i.e., abnormal wrist orientation and incorrect preshaping (Battaglini et al., 2002; Galletti et al., 2003). Lesions in the core regions of the dorsolateral pathway (AIP and F5p), have been reported to affect hand preshaping (i.e., grasping), leaving the reach component unaffected. After inactivation of AIP, monkeys showed abnormal hand preshaping during prehension (Gallese et al., 1994). The deficit was evident only, or mainly, when a precision grip was required, whereas whole hand prehension was generally unimpaired. This suggests that the potential impairment was evident only when more precise sensorimotor control was required. Inactivation of F5p (Fogassi et al., 2001) leads to a similar impairment with abnormal preshaping of the hand and wrist orientation, mainly evident while grasping small objects. Crucially, inactivation of the nearby F5 subarea (F5c), possessing the same visuomotor properties (Bonini et al., 2014), did not lead to any grasping deficits (Fogassi et al., 2001).

These results show that both pathways are *causally* involved in processing grasping, and also support a behavioral dissociation: lesions in the dorsolateral pathway impair mainly grasping, whereas damage within the dorsomedial pathway affects only reaching (MIP) or both reaching and grasping (V6A). If we link these results to neurophysiological findings, it is evident that the coding of both reaching and grasping within V6A has a clear behavioral relevance, possibly reflecting the processing of the whole act of prehension, integrating reaching and grasping information (Grafton, 2010; Fattori et al., 2010). The dorsolateral pathway (AIP and F5p) seems more strongly involved in coding visually guided grasping, particularly when this requires a high level of integration of visuospatial and contextual information for planning and controlling hand preshaping (Verhagen et al., 2008; Fattori et al., 2010).

It is more difficult to assess specific behavioral deficits based on human lesion studies, as the extent of brain damage is generally wider, encompassing more than a single cortical region. Nevertheless, recent studies support a similar account, with lesions in posterior PPC leading to reaching, and possibly also grasping deficits (Karnath and Perenin, 2005; Cavina-Pratesi et al., 2010a), and lesions in anterior PPC leading mainly to grasping impairments (Binkofski and Buxbaum, 2013).

Complementary information can be derived from “virtual lesion” TMS studies. This approach can inform us more accurately on where and at which stage (planning and/or online control) a temporary lesion affects grasp coding. The causal role of the dorsolateral pathway in coding grasp-related information has been demonstrated both for aIPS and PMv (Olivier et al., 2007). These studies demonstrated the specific role of aIPS in hand pre-shaping during visually guided prehension (Rice et al., 2006; Davare et al., 2007; Vesia et al., 2013) and during rapid online correction after object perturbation (Tunik et al., 2005; Rice et al., 2006).

A possible causal role of the dorsomedial pathway in grasp coding has been put forward on the basis of a dissociation between PMd and PMv in a visually guided grasp-to-lift task (Davare et al., 2006). TMS applied to PMv impaired hand preshaping, whereas TMS applied to PMd interfered only with lifting the object, as if the coupling between reaching and grasping was affected. These results seem to suggest that the dorsolateral pathway is *causally* involved in grasp coding, whereas the dorsomedial is *causally* involved in coding the interaction between the two components of prehension.

## Recent advances and future directions

One major limit in the description of grasp coding in monkeys consists in being primarily based on studies recording single cell activity. This description has a high temporal resolution allowing to map activity related to the different stages of prehension (planning, execution, online control), but it is difficult to understand how information is transferred to other cortical sites, as normally only one, or few nearby, areas are recorded simultaneously. A solution might be the widespread adoption of multielectrode and multiple site recordings which will help understanding the evolution of grasp coding within different regions.

As an example, Townsend et al. (2011) simultaneously investigated the neural response of AIP and F5 during a delayed motor task adopting a multivariate approach, i.e., trying to decode grip type and object orientation during planning. The analysis was based on multi-unit activity (MUA) which showed similar tuning as single-unit activity (SUA). Decoding of grip type or orientation alone showed significant above chance performance in both areas, with a preference of coding for grip type in F5 and for orientation in AIP. Decoding of grip type and orientation showed the best performance when combining data simultaneously recorded from the two regions, suggesting that they play complementary roles in grasp coding.

This study (Townsend et al., 2011) highlights the potential of multisite recordings in defining functional properties of simultaneously recorded regions. Moreover, it demonstrates that MUA conveys meaningful grasp information. Recent studies showed that also power modulations of Local Field Potentials (LFPs) code grip information both within F5 (Spinks et al., 2008) and IPL (comprising area AIP; Asher et al., 2007).

Stark and Abeles (2007) simultaneously recorded from PMv and PMd investigating reach and grasp coding, showing that it is possible to decode reach direction and grip type, and even their interaction, using SUA, LFPs and MUA (called multi-spike activity in this study) recorded from the same multiple electrodes. The limit of this study was that it pooled neural signal from PMd and PMv for decoding, so it is not possible to understand the specific role of each region in grasp and/or reach coding.

Taken together, these studies (Asher et al., 2007; Stark and Abeles, 2007; Spinks et al., 2008; Townsend et al., 2011) show that SUA, MUA, and LFPs convey grasp information. It is unclear, however, to which extent these measures play similar or different roles in the coding of grasping and in its integration with reaching. Furthermore, the decoding approach might be adopted not only to define the content, but also the different phases of prehension at which the coding of grasp information might happen, as recently shown for the early coding of observed graspable objects within AIP (Srivastava et al., 2009; Sakaguchi et al., 2010).

Monkey studies offer the unique possibility of obtaining a precise spatial and temporal map of the evolution of grasp coding, not only within one pathway but potentially within both. To explore the temporal relationship between coding within the two pathways and to test when and where grasping is integrated with reaching, future work might comprise simultaneous multisite recording (e.g., within AIP and V6A) during a grasping task. Reversible lesion studies might then be used to test the causal role of the same regions in the integration of the two components, identifying which signal (SUA, MUA, LFPs) or combination of signals conveys such integration.

Most of our knowledge on the human prehension system stems from neuroimaging data. Given the dynamic nature of prehension, it is crucial to understand the temporal evolution of its coding and of the interaction between grasping and reaching. fMRI lacks the temporal resolution needed for investigating these temporal aspects. In addition, it is difficult to understand when this integration would happen, as most fMRI studies did not separate between a planning and execution phase (but see Gallivan et al., 2011, 2013). A potential tool to unveil the neural dynamics of the integration of reach and grasp coding resides in exploiting high temporal resolution methods (EEG and MEG), which record signals more comparable with monkey neurophysiological data, particularly with LFPs.

A recent study started to tackle this issue by investigating prehension coding during planning using a combination of EEG, TMS and kinematic recordings (Verhagen et al., 2013). This study suggested a hierarchical organization of the two pathways, with the processing within the dorsomedial pathway being temporally dependent on aIPS activity. These results are further corroborated by another EEG study (Tunik et al., 2008) using a perturbation task, i.e., changing the orientation of the object during prehension. Adopting a different approach, i.e., microstate analysis, the results supported similar conclusions, showing two different processes after movement onset: one within aIPS and the other within posterior SPL. The process within the dorsomedial pathway was always following the one in the dorsolateral. This seems to suggest that aIPS is involved in integrating information for creating an action plan, whereas the activation of SPL was coincident with the start of the online adjustment, always following the end of aIPS recruitment.

These EEG results suggest that the two pathways interact during prehension coding and that the dorsolateral pathway could drive processing within the dorsomedial one. It is still unclear if this is the only type of interaction between the two pathways, or if other interactions can occur depending on task demands (e.g., level of online control, Grol et al., 2007) or between these two pathways and the ventral stream (Verhagen et al., 2008, 2012). Moreover, these EEG studies (Tunik et al., 2008; Verhagen et al., 2013; see also De Sanctis et al., 2013; Tarantino et al., 2014) demonstrate the potential of neurophysiological investigations as a tool for identifying potential time windows and cortical sites of integration, which could be subsequently tested adopting virtual lesions.

We have provided an up-to-date overview of the recent developments on grasp coding: at present we have a better understanding of *where* grasping (i.e., grip information) is coded and *which regions are causally involved* in its processing, but we still miss critical information about *when and where* this information is integrated with reaching (i.e., transport information). As described in the previous sections, integration between these two types of information might take place within both pathways at a functional level. By contrast, lesion studies seem to point to the integration of transport and grip information mainly within the dorsomedial pathway. How can we reconcile the discrepancy between these two levels of description?

Various accounts have proposed that the difference in coding characterizing the dorsolateral and the dorsomedial stream might emerge from a more general level of processing rather than from a distinction based on grasping and reaching (Rizzolatti and Matelli, 2003; Verhagen et al., 2008, 2012, 2013; Glover et al., 2012). Information about the temporal dynamics within the prehension system might be a critical factor to unravel these unsolved issues, permitting also to understand what type of information is driving the processing within these two pathways.

## Conflict of interest statement

The authors declare that the research was conducted in the absence of any commercial or financial relationships that could be construed as a potential conflict of interest.
